# Impact of primary site on survival in patients with nasopharyngeal carcinoma from 2004 to 2015

**DOI:** 10.3389/fsurg.2022.1001849

**Published:** 2022-11-04

**Authors:** Tianyi Shen, Wenting Cai, Tingting Li, Donghui Yu, Chengda Ren, Jing Yu

**Affiliations:** ^1^ Department of Ophthalmology, Shanghai Tenth People's Hospital, Tongji University, School of Medicine, Shanghai, China; ^2^ Department of Ophthalmology, The Third People’s Hospital of Bengbu, Bengbu, China

**Keywords:** nasopharyngeal neoplasms, SEER program, second primary, initial primary, prognosis

## Abstract

**Background:**

Nasopharynx carcinoma (NPC) is the most common malignant tumor of the nasopharynx. Many studies have shown some factors related with the prognosis of NPC patients. Our study aims to evaluate the differences of prognosis between initial and second primary NPC.

**Material and methods:**

The Surveillance, Epidemiology, and End Results (SEER) program was used to perform the population-based analysis in NPC patients who were newly diagnosed between 2004 and 2015. Kaplan-Meier and Cox regressions were used to evaluate the effects of primary site on the overall survival (OS), as well as the cancer-specific survival (CSS).

**Results:**

Our study included 5,012 NPC patients: 4,474 initial primary NPC patients and 5,38 s primary NPC patients. Significant differences were observed in sex, age at diagnosis, race, median household income, histological type, American Joint Committee on Cancer (AJCC) stage, N-stage, radiation treatment and chemotherapy between patients with initial and second NPC (*P *< 0.05). Moreover, the patients with second NPC had longer survival months. In addition, radiation and chemotherapy were recommended both in first and second primary NPC patients.

**Conclusion:**

Worse prognosis was observed in patients with second primary NPC compared with those with primary NPC in all subgroups of AJCC stage and age at diagnosis.

## Introduction

Nasopharynx carcinoma (NPC) is the most common malignant tumor of the nasopharynx, and this cancer is especially endemic in southeast Asian and Mediterranean populations (1). Other risk factors also include Epstein-Barr virus (EBV) exposure (2–4), environment (5, 6) and heavy alcohol intake (7, 8). This disease is often initially asymptomatic, and the first symptoms (nasal obstruction, sore throat, headache, epistaxis, and seromucous otitis) are non-specific. NPC is unfortunately commonly diagnosed once it is locally evolved, and greater than 80% of patients present with lymph node metastases at the time of the diagnosis. Based on the World Health Organization (WHO) histopathological grading system (9), there are three types of NPC including keratinizing squamous cell carcinoma, nonkeratinizing squamous cell carcinoma, and undifferentiated carcinoma.

Diagnosis is based on biopsy of the resected nasopharyngeal mass. Prior to biopsy, careful physical examination and fiberoptic endoscopic examination, evaluation of cranial nerve function, computed tomographic (CT) scan, magnetic resonance imaging (MRI) and EBV titers test should be performed (10). Detailed stage information for NPC is defined by the TNM system (11).

Given the proximity between nasopharyngeal cancer and essential risk organs, such as the brainstem and visual apparatus, treatment is complicated (10). Standard treatments for nasopharyngeal cancer patients include radiation therapy, concurrent chemoradiation followed by adjuvant chemotherapy, and surgery. Among these treatments, high-dose radiation therapy with chemotherapy is the primary treatment (12). Some new types of treatment for NPC are currently being tested in clinical trials. For example, monoclonal antibodies, such as nivolumab and ipilimumab, may interfere with the ability of tumor cells to grow and spread (13). For patients with EBV infection, a trial focused on EBV DNA-based individualized treatment is ongoing (14, 15). NPC treatments may cause side effects, such as anemia, appetite loss, alopecia, and lymphedema, which are also commonly reported in other cancer treatments.

According to our literature review, major factors affecting the prognosis of NPC include tumor size, and neck node involvement (16). Other factors potentially associated with the outcome of treatment include age, pregnancy, immunodeficiency, incomplete excision of involved neck nodes, and locoregional relapse, are still under study. The purpose of this article is to assess the prognosis differences between patients of initial and second primary NPC.

## Material and methods

### Study populations

The Surveillance, Epidemiology, and End Results (SEER) database was applied to extract the NPC patients who were diagnosed range from 2004 to 2015. In the present study, we use SEER∗Stat 8.3.5 software (National Cancer Institute, Bethesda, MD) to collect the initial data. We obtained detailed data on patients diagnosed with NPC between 2004 and 2015 from SEER-18. We excluded cases using the following criteria: (a) survival time unknown; (b) initial and primary NPC patients with incomplete data; (c) age at diagnosis under 18 years old; (d) data with unknown AJCC and T/N/M stage.

### Study variables

Several clinicopathological characteristics were included in our study. Gender was divided into male and female patients. Race was separated into white, black, and unknown. Patients diagnosed in different age were classified into three groups: younger than 45, between 45 and 65, older than 65. Marital status was divided into married, unmarried, and unknown. We also included metropolitan and nonmetropolitan. Median household income was separated into quartiles, quartile I was <$35,000, quartile II was $35,000–$45,000, quartile III was $45,001–$55,000, and quartile IV was >$55,000, respectively. Histology type included squamous cell carcinoma or others. Other indexes such as AJCC stage, T-stage, N-stage, M-stage were divided according to their grades. We considered overall survival (OS) and cancer-specific survival (CSS) as primary outcomes.

### Ethical approval and consent

Human participants involved in this study were all subject to the ethical standards of the institutional research committee, and to the 1964 Helsinki declaration as well as its later amendments or comparable ethical standards. No animal studies were included in present study.

### Statistical analysis

All analyses were performed using GraphPad Prism version 6.01and IBM SPSS Statistics version 20, the level of statistical significance was considered as *P < *0.05. Patients were followed up until December 2015. OS and CSS served as the primary outcomes. In this study, the relevant factors included gender, race, marital status, urban or rural region, age at diagnosis, histology type, median household income, AJCC as well as TNM stage, and treatment including surgery, radiation and chemotherapy. Data are presented as the mean ± SD. Categorical variables were recorded as counts (percentage). Analysis of variance was used to compare continuous variables with symmetric distributions across primary and second NPC subgroups. The Kaplan–Meier method was used to plot the survival distributions. Chi square tests were used to compare categorical variables between subgroups. Next, we conducted a multivariable Cox proportional hazard analysis to obtain hazard ratios (HR) and 95% CI in accordance with demographic and clinical covariates.

## Results and analysis

### Patient baseline characteristics

**Figure 1:**
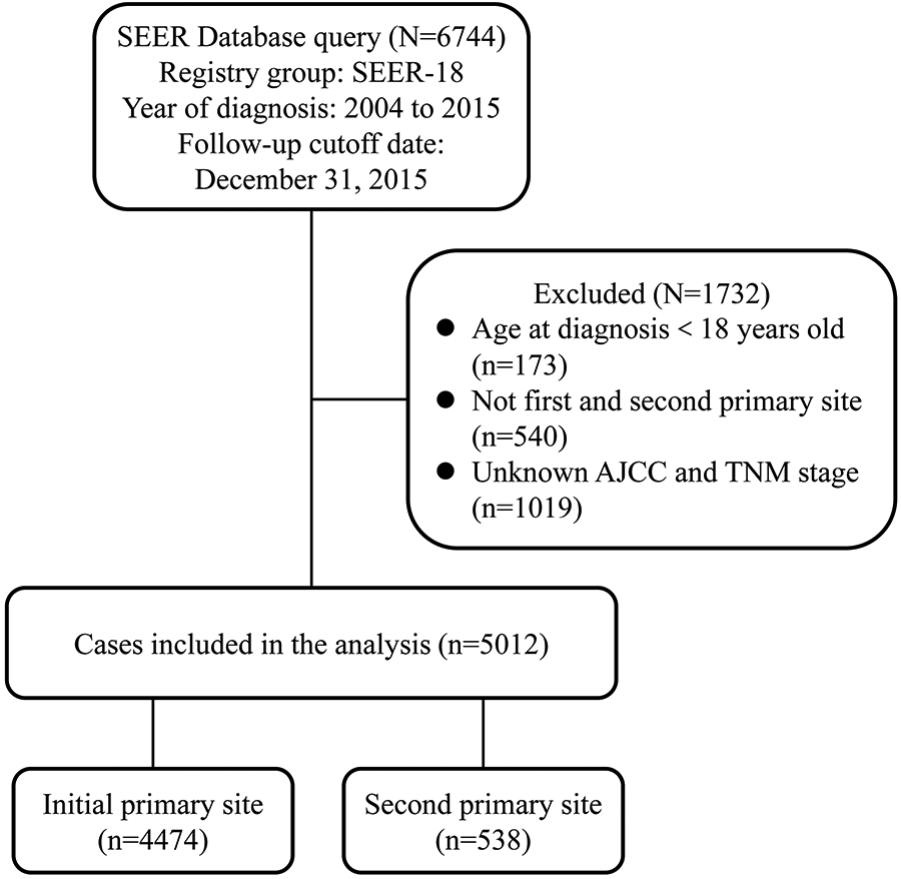
Flowchart describing the inclusion and exclusion of patients.

We extracted data on 6,744 patients diagnosed with NPC between 2004 and 2015 from SEER-18. According to the schematic diagram of Figure 1, 5,012 eligible patients were included. For the primary comparison, the original dataset (*n* = 5,012) included 4,474 initial NPC patients and 5,38 s primary NPC patients. Significant differences were observed in gender, age at diagnosis, race, median household income, histology type, AJCC stage, N stage, radiation treatment and chemotherapy between these two groups. The majority of cases in our cohort were 45–64 years of age (51.4%), white (47.4%), married (58.6%), nonmetropolitan (83.1%) and histology type of squamous cell carcinoma (59.5%). Second primary NPC patients had higher ratios in diagnosed age over 65 years (52.8% vs. 23.1%) and squamous cell carcinoma (71.0% vs. 58.1%) than initial cases (Table 1).

**Table 1:** Demographic and clinical characteristics of NPC patients.

Characteristic	Total	Initial NPC	Second NPC	*P*
		*N* (Percentage)	*N* (Percentage)	
Total	5,012	4,474 (89.3%)	538 (10.7%)	
**Gender**				0.003
Male	3,493 (69.7%)	3,148 (70.4%)	345 (64.1%)	
Female	1,519 (30.3%)	1,326 (29.6%)	193 (35.9%)	
**Race**				0.000
White	2,376 (47.4%)	2,031 (45.4%)	345 (64.1%)	
Black	590 (11.8%)	524 (11.7%)	66 (12.3%)	
Unknown	2,046 (40.8%)	1,919 (42.9%)	127 (23.6%)	
**Age at diagnosis**				0.000
<45	1,121 (22.4%)	1,105 (24.7%)	16 (3.0%)	
45–64	2,575 (51.4%)	2,337 (52.2%)	238 (44.2%)	
≥65	1,316 (26.3%)	1,032 (23.1%)	284 (52.8%)	
**Marital status**				0.582
No	1,817 (36.3%)	1,619 (36.2%)	198 (36.8%)	
Yes	2,939 (58.6%)	2,631 (58.8%)	308 (57.2%)	
Unknown	256 (5.1%)	224 (5.0%)	32 (5.9%)	
**Urban-rural residence**				0.062
Metropolitan	400 (8.0%)	346 (7.7%)	54 (10.0%)	
Nonmetropolitan	4,612 (92.0%)	4,128 (92.3%)	484 (90.0%)	
**Median household income**				0.001
Quartile I	601 (12.0%)	526 (11.8%)	75 (13.9%)	
Quartile II	1,674 (33.4%)	1,474 (32.9%)	200 (37.2%)	
Quartile III	1,209 (24.1%)	1,068 (23.9%)	141 (26.2%)	
Quartile IV	1,528 (30.5%)	1,406 (31.4%)	122 (22.7%)	
**Histology Type**				0
Squamous cell carcinoma	2,981 (59.5%)	2,599 (58.1%)	382 (71.0%)	
Others	2,031 (40.5%)	1,875 (41.9%)	156 (29.0%)	
**AJCC stage**				0
I	464 (9.3%)	376 (8.4%)	88 (16.4%)	
II	1,137 (22.7%)	1,010 (22.6%)	127 (23.6%)	
III	1,484 (29.6%)	1,334 (29.8%)	150 (27.9%)	
IV	1,927 (38.4%)	1,754 (39.2%)	173 (32.2%)	
**T stage**				0.411
0	16 (0.3%)	14 (0.3%)	2 (0.4%)	
1	1,584 (31.6%)	1,396 (31.2%)	188 (34.9%)	
2	1,214 (24.2%)	1,093 (24.4%)	121 (22.5%)	
3	1,047 (20.9%)	933 (20.9%)	114 (21.2%)	
4	1,151 (23.0%)	1,038 (23.2%)	113 (21.0%)	
**N stage**				0
0	1,358 (27.1%)	1,124 (25.1%)	234 (43.5%)	
1	1,642 (32.8%)	1,481 (33.1%)	161 (29.9%)	
2	1,374 (27.4%)	1,277 (28.5%)	97 (18.0%)	
3	638 (12.7%)	592 (13.2%)	46 (8.6%)	
**M stage**				0.726
0	4,487 (89.5%)	4,003 (89.5%)	484 (90.0%)	
1	525 (10.5%)	471 (10.5%)	54 (10.0%)	
**Surgery**				0.065
Performed	4,442 (88.6%)	496 (11.1%)	74 (13.8%)	
None or refused	570 (11.4%)	3,978 (88.9%)	464 (86.2%)	
**Radiation**				0
Yes	1,323 (26.4%)	1,217 (27.2%)	106 (19.7%)	
No	3,689 (73.6%)	3,257 (72.8%)	432 (80.3%)	
**Chemotherapy**				0
Yes	3,951 (78.8%)	3,611 (80.7%)	340 (63.2%)	
No	1,061 (21.2%)	863 (19.3%)	198 (36.8%)	

NPC, nasopharyngeal carcinoma.

A cohort of 5,012 patients with first and second primary nasopharyngeal carcinoma was included in the study. Patients who died from any cause were more likely to exhibit the following characteristics: second primary carcinoma (57.6%), males (41.4%), older patients (59.0%), unmarried patients (47.3%), metropolitan (49.5%), lower household income (51.9%), squamous cell carcinoma (43.5%), and higher grade of AJCC and TNM stage. Moreover, patients without treatments, including all types of surgeries, radiation and chemotherapy, were more likely to be deceased. Moreover, when analyzed by CSS, 25.8% of patients died from nasopharyngeal cancer, and the results from OS analysis kept the same (Table 2).

**Table 2:** Univariate analysis of all-cause and NPC-specific mortality for NPC patients.

Characteristic	All-cause mortality		*P*	NPC-specific death		*P*
	Dead	Alive		Dead	Alive	
	*N* (percentage)	*N* (percentage)		*N* (percentage)	*N* (percentage)	
Total	1,998 (39.9%)	3,014 (60.1%)		1,046 (25.8%)	3,014 (74.2%)	
**Primary site**			0.000			0.001
Initial NPC	1,688 (37.7%)	2,786 (62.3%)		947 (25.4%)	2,786 (74.6%)	
Second NPC	310 (57.6%)	228 (42.4%)		117 (33.9%)	228 (66.1%)	
**Gender**			0.001			0.004
Male	1,447 (41.4%)	2,046 (58.6%)		773 (27.4%)	2,046 (72.6%)	
Female	551 (36.3%)	968 (63.7%)		291 (23.1%)	968 (76.9%)	
**Age at diagnosis**			0.000			0.000
<45	263 (23.5%)	858 (76.5%)		171 (16.6%)	858 (83.4%)	
45–64	958 (37.2%)	1,617 (62.8%)		543 (25.1%)	1,617 (74.9%)	
≥65	777 (59.0%)	539 (41.0%)		350 (39.4%)	539 (60.6%)	
**Race**			0.000		0	
White	1,100 (46.3%)	1,276 (53.7%)		521 (29.0%)	1,276 (71.0%)	
Black	272 (46.1%)	318 (53.9%)		125 (28.2%)	318 (71.8%)	
Unknown	626 (30.6%)	1,420 (69.4%)		418 (22.7%)	1,420 (77.3%)	
**Marital status**			0.000			0.000
No	859 (47.3%)	958 (52.7%)		429 (30.9%)	958 (69.1%)	
Yes	1,043 (35.5%)	1,896 (64.5%)		585 (23.6%)	1,896 (62.9%)	
Unknown	96 (37.5%)	160 (62.5%)		50 (23.8%)	160 (76.2%)	
**Urban-rural residence**			0.000			0.052
Metropolitan	198 (49.5%)	202 (50.5%)		88 (30.3%)	202 (69.7%)	
Nonmetropolitan	1,800 (39%)	2,812 (61.0%)		976 (25.8%)	2,812 (74.2%)	
**Median household income**			0.000			0.000
Quartile I	312 (51.9%)	289 (48.1%)		144 (33.3%)	289 (66.7%)	
Quartile II	694 (41.5%)	980 (58.5%)		358 (26.8%)	980 (73.2%)	
Quartile III	500 (41.4%)	709 (58.6%)		273 (27.8%)	709 (72.2%)	
Quartile IV	492 (32.2%)	1,036 (67.8%)		289 (21.8%)	1,036 (78.2%)	
**Histology Type**			0.000			0.000
Squamous cell carcinoma	1,297 (43.5%)	1,684 (56.5%)		679 (28.7%)	1,684 (71.3%)	
Others	701 (34.5%)	1,330 (65.5%)		385 (22.4%)	1,330 (77.6%)	
**AJCC stage**			0.000			0.000
I	126 (27.2%)	338 (72.8%)		42 (11.1%)	338 (88.9%)	
II	328 (28.8%)	809 (71.2%)		147 (15.4%)	809 (84.6%)	
III	541 (36.5%)	943 (63.5%)		293 (23.7%)	943 (76.3%)	
IV	1,003 (52.0%)	924 (48.0%)		582 (38.6%)	924 (61.4%)	
**T stage**			0.000			0.000
0	10 (62.5%)	6 (37.5%)		3 (33.3%)	6 (66.7%)	
1	475 (30.0%)	1,109 (70.0%)		242 (17.9%)	1,109 (81.1%)	
2	433 (35.7%)	781 (64.3%)		217 (21.7%)	781 (78.3%)	
3	497 (47.5%)	550 (52.5%)		273 (33.2%)	550 (66.8%)	
4	583 (50.7%)	568 (49.3%)		329 (36.7%)	568 (63.3%)	
**N stage**			0.000			0.000
0	581 (42.8%)	777 (57.2%)		262 (25.2%)	777 (74.8%)	
1	593 (36.1%)	1,049 (63.9%)		319 (23.3%)	1,049 (76.7%)	
2	518 (37.7%)	856 (62.3%)		297 (25.8%)	856 (74.2%)	
3	306 (48.0%)	332 (52.0%)		186 (35.9%)	332 (64.1%)	
**M stage**			0.000			0.000
0	1,621 (36.1%)	2,866 (63.9%)		826 (22.4%)	2,866 (77.6%)	
1	377 (71.8%)	148 (28.2%)		238 (61.7%)	148 (38.3%)	
**Surgery**			0.000			0.000
Performed	177 (31.1%)	393 (68.9%)		79 (16.7%)	393 (83.3%)	
None or refused	1,821 (41.0%)	2,621 (59.0%)		985 (27.3%)	2,621 (72.7%)	
**Radiation**			0.000			0.000
Yes	425 (32.1%)	898 (67.9%)		238 (21.0%)	898 (29.8%)	
No	1,573 (42.6%)	2,116 (57.4%)		826 (28.1%)	2,116 (70.2%)	
**Chemotherapy**			0.000			0.000
Yes	1,441 (36.5%)	2,510 (63.5%)		820 (24.6%)	2,510 (75.4%)	
No	557 (52.5%)	504 (47.5%)		244 (32.6%)	504 (67.4%)	

NPC, nasopharyngeal carcinoma.

### Comparison of os and CSS between initial primary and second primary NPC patients

**Figure 2:**
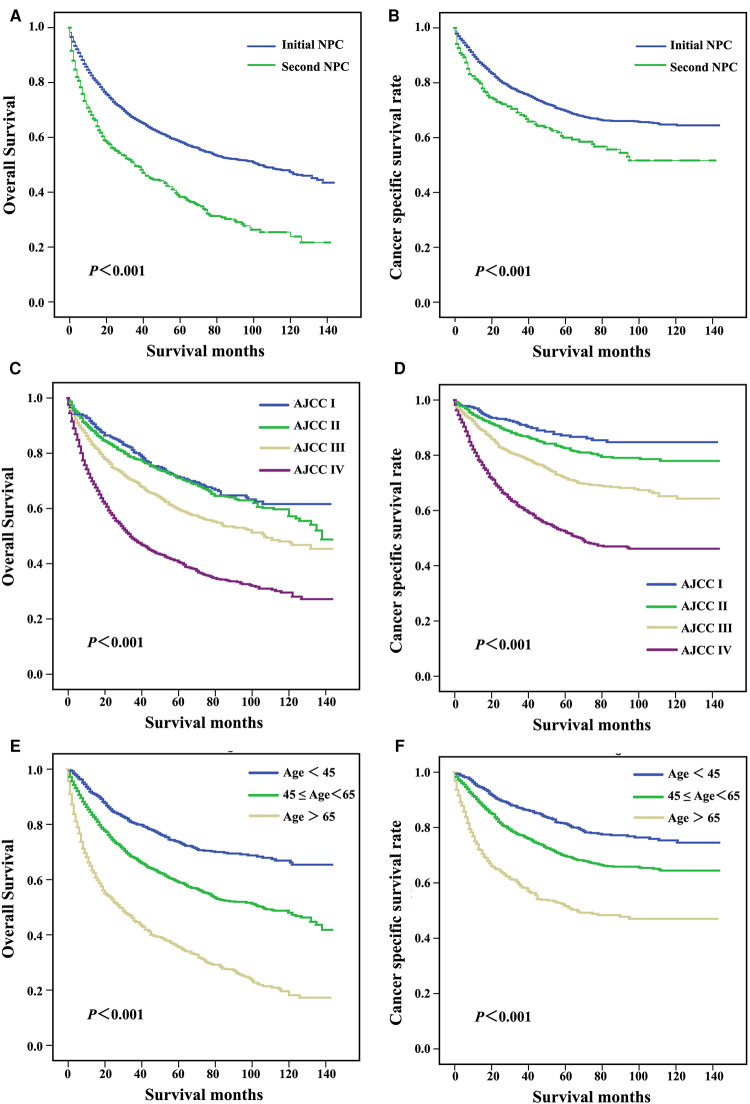
Survival curves for patients with different primary sites (**A,B**), AJCC stage (**C,D**), and age at diagnosis (**E,F**).

Log-rank tests for the OS and cause-specific Kaplan-Meier survival curves (Figure 1) revealed significant differences among the primary sites, AJCC grade and age in diagnosis groups. The OS and CSS of patients with initial NPC were significantly increased compared with patients with second NPC (62.3% vs. 42.4%, *P *< 0.001, Figure 2A; 74.6% vs. 66.1%, *P *< 0.001, Figure 2B, respectively). As shown in Figures 2C,D, OS and CSS of patients with low AJCC grades were significantly increased compared with patients with a high AJCC grade (*P *< 0.001). Patients whose age at diagnosis was greater than 65 exhibited significantly lower OS and significantly (both *P *< 0.001, Figures 2E,F).

**Figure 3:**
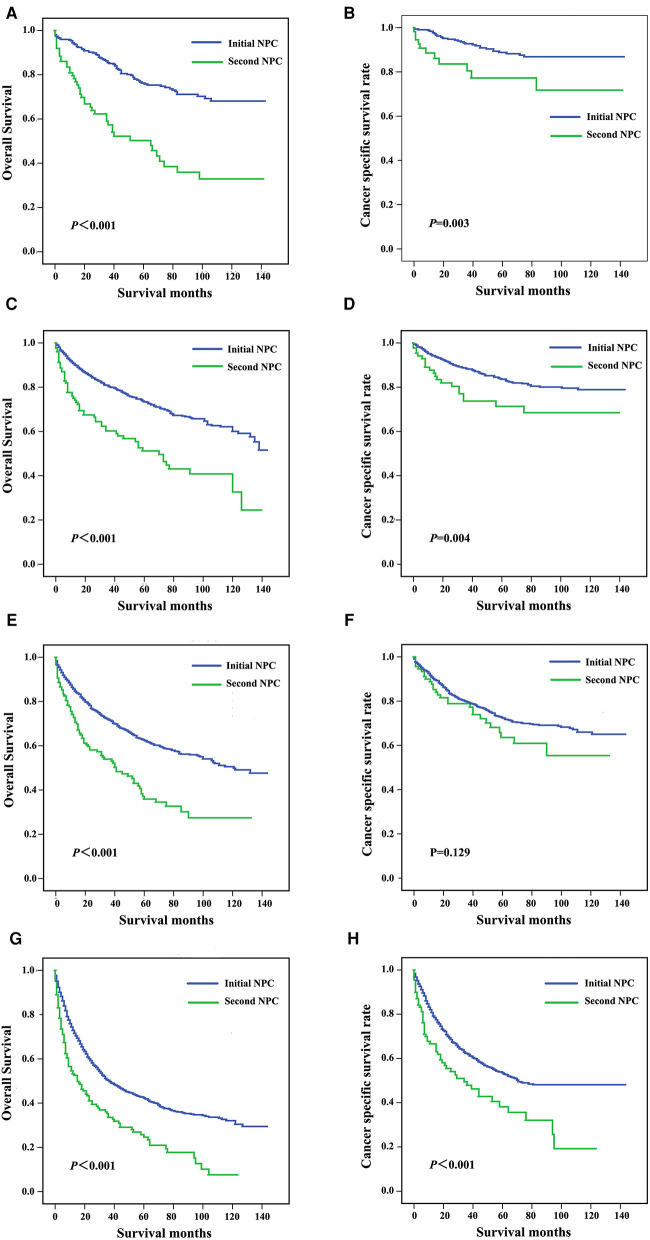
Survival curves for patients subgrouped by AJCC stage, including stage I (**A,B**), stage II (**C,D**), stage III (**E,F**), and stage IV (**G,H**).

**Figure 4:**
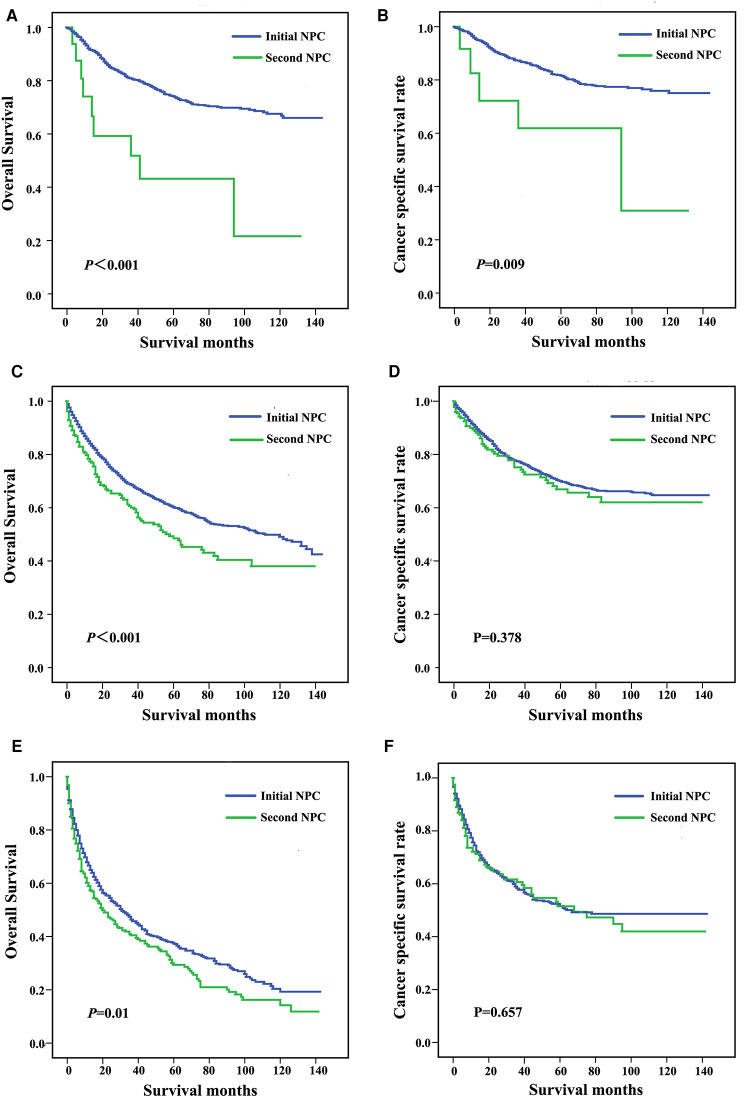
Survival curves for patients stratified by age at diagnosis, including <45 years (**A,B**), 45-64 years (**C,D**), and ≥65 years (**E,F**).

Patients were subgrouped according to AJCC stage and age at diagnosis. As shown in Figures 3A,B, patients diagnosed with AJCC I stage and patients with second NPC exhibited increased overall and cancer-specific mortality, and similar findings were noted for AJCC II to IV stage patients. When analyzed by both OS and CSS, the prognosis of second primary NPC was poorer compared with first primary NPC regardless of AJCC grade. As shown in Figure 4, all subgroups of age at diagnosis exhibited consistent results, and patients with second primary carcinoma were more likely to exhibit increased all-cause mortality. However, no significant differences were between the first and second primary sites when patients were diagnosed between 45 and 65 (*P *> 0.05, Figure 4D) and >65 years (*P *> 0.05, Figure 4F) based on CSS analysis.

### Univariate and multivariate analysis of factors associated with all-cause mortality and cancer-specific mortality

We performed univariate and multivariate Cox regression analyses to analyze the prognostic factors (Table 3). When analyzed by OS, the HR of second NPC was 1.231, and 95% CI was 1.084 to 1.398 (*P *< 0.001) compared with patients with initial NPC. Increased risks were associated with all-cause mortality in multivariate analysis. According to the univariate analysis, primary sites of second NPC were factors that affected cancer-specific mortality (HR = 1.517, *P *< 0.001) when referred to CSS. In addition, male (*P *< 0.05), age over 65 years old at diagnosis (*P *< 0.001), black race (*P *< 0.01), and histology type of squamous cell (*P *< 0.001) were all risk factors that affected not only all-cause mortality, but also cancer-specific mortality. AJCC stage, T stage and M stage were also closely associated with overall as well as cancer-specific mortality (all *P *< 0.05), whereas N stage did not influence cancer-specific mortality (*P *> 0.05). According to multivariate logistic regression for cancer-specific mortality, AJCC stage IV had the highest mortality among all the stages, and the HR was 4.304, with 95% CI ranging from 2.937 to 6.307 when compared with stage I.

**Table 3:** Univariate and multivariate analysis for evaluating prognosis factors on mortality.

	Overall mortality				Cancer-specific mortality			
	Univariate Analysis		Multivariate Analysis		Univariate Analysis		Multivariate Analysis	
	HR (95% CI)	*P*	HR (95% CI)	*P*	HR (95% CI)	*P*	HR (95% CI)	*P*
**Primary site**		0		0.001		0		0.816
Initial NPC	1		1		1		NA	
Second NPC	1.863 (1.650–2.103)		1.231 (1.084–1.398)		1.517 (1.252–1.838)		NA	
**Gender**		0.002		0.002		0.006		0.023
Male	1		1		1		1	
Female	0.853 (0.773–0.941)		0.854 (0.773–0.943)		0.828 (0.724–0.947)		0.854 (0.745–0.978)	
**Age at diagnosis**		0		0		0		0
<45	1		1		1		1	
45–64	1.808 (1.577–2.073)		1.667 (1.450–1.915)		1.683 (1.417–1.999)		1.653 (1.388–1.967)	
≥65	3.891 (3.381–4.477)		3.339 (2.881–3.871)		3.477 (2.894–4.177)		3.373 (2.793–4.073)	
**Race**		0		0		0		0.002
White	1		1		1		1	
Black	1.031 (0.903–1.178)		1.056 (0.923–1.209)		1.001 (0.824–1.217)		1.054 (0.865–1.285)	
Unknown	0.581 (0.527–0.641)		0.670 (0.605–0.742)		0.733 (0.645–0.834)		0.806 (0.705–0.920）	
**AJCC stage**		0		0		0		0
I	1		1		1		1	
II	1.092 (0.890–1.342)		1.601 (1.248–2.054)		1.444 (1.025–2.035)		2.107 (1.456–3.049)	
III	1.547 (1.274–1.878)		2.098 (1.614–2.727)		2.438 (1.764–3.369)		3.231 (2.230–4.683)	
IV	2.830 (2.350–3.408)		2.425 (1.827–3.219)		4.929 (3.602–6.744)		4.304 (2.937–6.307)	
**Histology Type**		0		0		0		0
Squamous cell carcinoma	1		1		1		1	
Others	0.691 (0.631–0.758)		0.806 (0.733–0.886)		0.695 (0.613–0.787)		0.792 (0.696–0.900)	
**T stage**		0		0		0		0
0	1		1		1		1	
1	0.387 (0.207–0.724)		0.920 (0.484–1.750)		0.615 (0.197–1.920)		1.938 (0.612–6.136)	
2	0.484 (0.258–0.906)		1.051 (0.554–1.997)		0.766 (0.245–2.392)		2.142 (0.677–6.778)	
3	0.760 (0.406–1.421)		1.404 (0.740–2.665)		1.349 (0.432–4.208)		3.041 (0.961–9.619)	
4	0.918 (0.491–1.716)		1.580 (0.833–2.995)		1.684 (0.540–5.249)		3.182 (1.011–10.020)	
**N stage**		0		0.015		0		0.051
0	1		1		1		NA	
1	0.805 (0.718–0.902)		0.979 (0.858–1.117)		0.907 (0.771–1.068)		NA	
2	0.865 (0.768–0.974)		0.956 (0.826–1.107)		1.023 (0.867–1.208)		NA	
3	1.302 (1.133–1.495)		1.264 (1.055–1.515)		1.639 (1.358–1.979)		NA	
**M stage**		0		0		0		0
0	1		1		1		1	
1	3.206 (2.862–3.591)		2.576 (2.219–2.991)		4.244 (3.668–4.910)		2.874 (2.384–3.465)	
**Surgery**		0		0		0		0
Performed	1		1		1		1	
None or refused	1.522 (1.304–1.776)		1.499 (1.248–1.799)		1.846 (1.468–2.322)		1.780 (1.405–2.255)	
**Radiation**		0		0.046		0		0.379
Yes	1		1		1		NA	
No	1.502 (1.350–1.672)		1.138 (1.002–1.292)		1.477 (1.279–1.706)		NA	
**Chemotherapy**		0		0		0		0
Yes	1		1		1		1	
No	1.845 (1.673–2.035)		2.331 (2.088–2.602)		1.538 (1.333–1.775)		2.394 (2.053–2.793)	

In the stratified log-rank test, the overall mortality and cancer-specific mortality for age at diagnosis, AJCC stage, and primary site were 493.836, 352.393 and 105.772, respectively, when analyzed for all causes of death. Similar results were noted for the previously described variables. Chi-square of OS and CSS for factors, such as gender and urban-rural residence, revealed less influence compared with the other variables (Supplementary Figure S1).

### Comparison of different therapies for initial and second primary NPC

**Figure 5:**
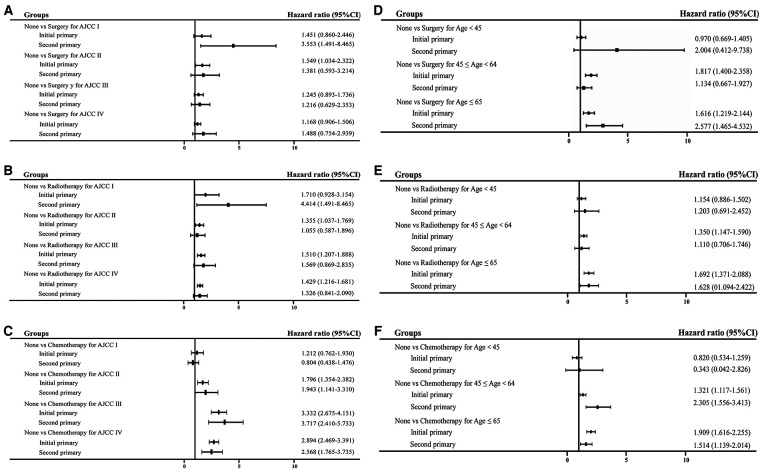
Forest plot of HR and 95% CI in overall mortality between initial and second primary patients stratified by different subgroups. (**A**) Comparison of surgery performance in different AJCC stage; (**B**) comparison of radiation performance in different AJCC stage; (**C**) comparison of chemotherapy treatment in different AJCC stage; (**D**) comparison of surgery performance in different age at diagnosis; (**E**) comparison of radiation performance in different age at diagnosis; (**F**) comparison of chemotherapy treatment in different age at diagnosis.

To analyze better therapies for initial and second NPC at different AJCC stages and age at diagnosis, forest plot was performed to clearly observe the comparison. As shown in Figure 5, surgery, radiation and chemotherapy were applied to initial and second primary NPC patients. Among nonsurgical second primary stage I patients, the overall mortality increased significantly (HR = 2.553, 95% CI = 1.491–8.465). Similar results were noted for patients at different ages of diagnosis. Thus, both the initial and second primary patients could benefit from the prompt therapies.

## Discussion

In our study, we used SEER data to assess the interaction between TNM stage, AJCC stage, primary site, histology type, marital status, race, age at diagnosis, and survival in patients with NPC. Patients with second primary NPC suffered worse prognosis in comparison with the initial primary NPC group. Our study showed a significant survival benefit for patients younger than 45 years of age as well, regardless of the primary tumor site. As age increased, the prognosis tended to become worse. Moreover, in each age group, the initial NPC subgroup exhibited a better prognosis compared with the second NPC group. A study showed the negative effect of second primary malignancies, including the occurrence of NPC, on the survival rate of 93,891 patients who suffered from head and neck cancer (17). In addition, it is thought-provoking that in other tumors, such as lung and colorectal cancer, the mortality of the second primary cancer was higher than that of the initial (18, 19). These survival patterns are fundamentally consistent with our literature review. The existence of this situation is complex and multifactorial. Chen et al. found that the longer the interval between the second and initial primary cancer, the higher the morbidity and mortality of the second primary cancer occurred in a 25-year study in Taiwan (20). Kong et al. reported that the morbidity of second primary tumors tended to increase after definitive radiation therapy. Older patients with NPC (age ≥ 50 years) may be at increased risk (21). Chemotherapy and radiation therapy as well as some other types of cancer treatment may increase the risk of a second primary cancer. The presence of an initial primary cancer may also indicate some inherited gene mutations or existing exposure to cancer-causing factors, such as alcohol and tobacco, thus, the risk of second primary cancer increases. Overall, advanced age is the most important risk factor for most cancers (22). In addition to increased susceptibility to cancers, aging patients also commonly exhibit other diseases, leading to a weak physical condition that confers fragility when facing extra health problems and poorer long-term survival. A small discrepancy was noted in the groups of patients >45 years of age. Specifically, differences in CSS rates between initial and second primary NPC were not markable. This finding may be attributed to the fact that as patients age, more patients die as a result of factors other than NPC, especially in consideration of the aforementioned physical characteristics of aging people.

Female was observed as a weak protective factor for NPC *via* multivariate analysis. Compared with cases with unknown race information, a notable survival benefit was observed in African and Caucasian patients. Similar results were found both in our study and a previous study, which indicated that survival of initial NPC exhibited no preference for any gender or race (23). However, the concentration on race- or gender-related survival benefits of previous research in second NPC is lacking. NPCs most commonly start in the squamous cells lining the nasopharynx (24). The squamous cell subtype of NPC, which was reported as the major type in both initial and second primary NPC cases, was associated with a less favorable prognosis in our study. Baxi SS et al. reported that many survivors afflicted with head and neck squamous cell carcinomas ultimately died from cancers other than these malignancies and/or noncancer causes (25). The primary site may not significantly affect the prognosis of squamous cell NPCs.

The risk of death was steadily augmented based on elevated tumor T-stage except for T0, and the lethal risk of M1 stage cases dramatically exceeded that of M0. In contrast, such a clear trend was not noted in patients of different N-stage subgroups. Risk of N3 stage noticeably outweighed N0, N1, and N2, but the risks in the three latter stages were not distinguishable. Primary NPC is known for its high metastatic potential, primarily to lymph nodes, *via* TGF-β/SMAD signaling and Snail/TEL2 pathways (26, 27). Malignancy of tumors exclusively involving adjacent lymph nodes may not conspicuously influence the differences in NPC prognosis. Initial NPC exhibited an incidence roughly equivalent to that of second NPC for each grade of T, N, or M stage. With respect to the composition of T0 group in our dataset (only 16 cases in total and 9 out of them were M1, which meant grouping by one dimension perhaps interrelated with others) and based on clinical experience, we analyzed the AJCC stage of cases. As the stage changed from I to IV, the risk of death significantly increased, ideally displaying the consistency between clinic prognosis and AJCC staging. In each AJCC stage group, the initial NPC subgroup exhibited a more favorable prognosis compared with the second NPC subgroup, indicating that primary site may be an independent risk factor beyond AJCC staging, and second primary malignancies might be a complex condition for current staging systems.

Prominent benefits were also noted for patients receiving standardized treatments compared with untreated. Any type of surgery, radiation, or chemotherapy that was appropriately performed significantly reduced mortality regardless of the primary site. Given that radiation therapy in localized head and neck cancers decreased the incidence of second head and neck cancer cancers, the reason for the better prognosis may be complicated (28). This finding indicates that once NPC is diagnosed, the patient should initiate available treatments as recommended as soon as possible.

The survival trends in SEER datasets in this study are generally consistent with the published literature, and statistical analysis of different aspects revealed compatible results. These results demonstrated that our findings were robust. Although SEER has collected information for cancer statistics on a large number of patients in the United States, NPC is still a rare tumor in this database considering the significant difference in prevalence among races and districts. Given that certain information is limited in SEER datasets, grouping criteria may not be definite and may require more deliberated discussion. For example, subgroups of the histological type “non-squamous cell carcinoma” and age could be stratified using a more detailed method, which may help adjust the accuracy of our conclusions. Additionally, the factor of interval between the index cancer and the second primary cancer was not included in this study. Patients with metachronous second primary head and neck cancers exhibit a better prognosis compared with those who present with synchronous lesions, implying the possibility of survival variance between early and late second NPCs (29–31). Tobacco and alcohol use in NPC survivors was not discussed here but has been highlighted as a threat to survival rates of cancer patients (32–34). Given these limitations, future studies are warranted to confirm the observations reported here.

## Conclusion

In conclusion, our study indicated that patients with second primary NPC exhibited a worse prognosis compared with those with initial NPC in all AJCC stage and age at diagnosis subgroups. Despite the primary site, NPC patients could benefit from standardized treatments, including surgery, radiation and chemotherapy.

## Data Availability

The original contributions presented in the study are included in the article/**Supplementary Material**, further inquiries can be directed to the corresponding author/s.
