# Supplementary figures and images for: Impact of primary site on survival in patients with nasopharyngeal carcinoma from 2004 to 2015
Source: Front Surg. 2022 Nov 4;9:1001849. doi: 10.3389/fsurg.2022.1001849 (PMC9671952; doi:10.3389/fsurg.2022.1001849)

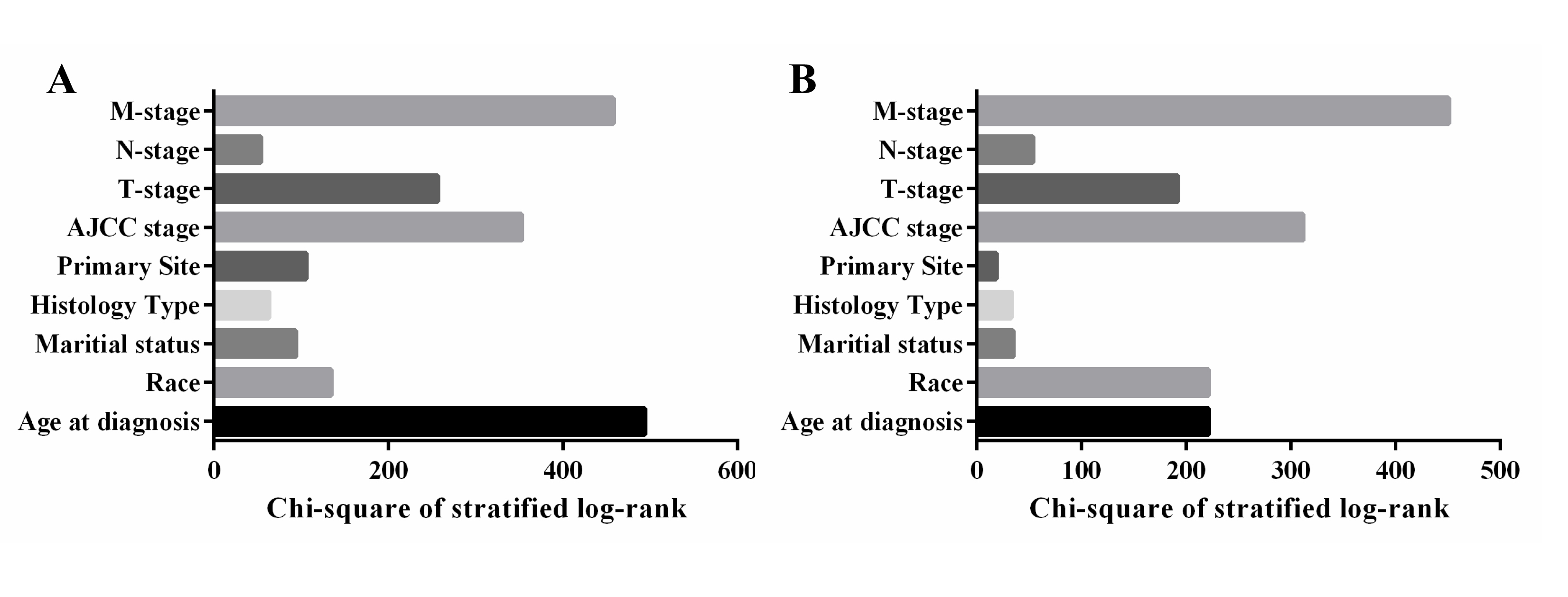

Supplement: Supplementary file 1 [file Image1.jpeg]
